# Post-COVID-19 Pain Is Not Associated with DNA Methylation Levels of the *ACE2* Promoter in COVID-19 Survivors Hospitalized Due to SARS-CoV-2 Infection

**DOI:** 10.3390/biomedicines12081662

**Published:** 2024-07-25

**Authors:** César Fernández-de-las-Peñas, Gema Díaz-Gil, Antonio Gil-Crujera, Stella M. Gómez-Sánchez, Silvia Ambite-Quesada, Anabel Franco-Moreno, Pablo Ryan-Murua, Juan Torres-Macho, Oscar J. Pellicer-Valero, Lars Arendt-Nielsen, Rocco Giordano

**Affiliations:** 1 Department of Physical Therapy, Occupational Therapy, Rehabilitation and Physical Medicine, Universidad Rey Juan Carlos (URJC), 28922 Alcorcón, Spain; silvia.ambite.quesada@urjc.es; 2 Center for Neuroplasticity and Pain (CNAP), Sensory Motor Interaction (SMI), Department of Health Science and Technology, Faculty of Medicine, Aalborg University, DK-9220 Aalborg, Denmark; lan@hst.aau.dk (L.A.-N.); rg@hst.aau.dk (R.G.); 3 Research Group GAMDES, Department of Basic Health Sciences, Universidad Rey Juan Carlos (URJC), 28933 Madrid, Spain; gema.diaz@urjc.es (G.D.-G.); antonio.gil@urjc.es (A.G.-C.); stella.gomez@urjc.es (S.M.G.-S.); 4 Department of Internal Medicine, Hospital Universitario Infanta Leonor-Virgen de la Torre, 28031 Madrid, Spain; anaisabel.franco@salud.madrid.org (A.F.-M.); pablo.ryan@salud.madrid.org (P.R.-M.); juan.torresm@salud.madrid.org (J.T.-M.); 5 Department of Medicine, School of Medicine, Universidad Complutense de Madrid, 28040 Madrid, Spain; 6 Image Processing Laboratory (IPL), Universitat de València, Parc Científic, 46980 Paterna, Spain; oscar.pellicer@uv.es; 7 Department of Gastroenterology & Hepatology, Mech-Sense, Clinical Institute, Aalborg University Hospital, DK-9000 Aalborg, Denmark; 8 Steno Diabetes Center North Denmark, Clinical Institute, Aalborg University Hospital, DK-9000 Aalborg, Denmark; 9 Department of Oral and Maxillofacial Surgery, Aalborg University Hospital, DK-9000 Aalborg, Denmark

**Keywords:** methylation, *ACE2*, pain, post-COVID-19, long COVID

## Abstract

One of theories explaining the development of long-lasting symptoms after an acute severe acute respiratory syndrome coronavirus 2 (SARS-CoV-2) infection include changes in the methylation pattern of the host. The current study aimed to investigate whether DNA methylation levels associated with the angiotensin-converting enzyme 2 (*ACE2*) promoter are different when comparing individuals previously hospitalized due to COVID-19 who then developed long-lasting post-COVID pain with those previously hospitalized due to COVID-19 who did not develop post-COVID-19 pain symptoms. Non-stimulated saliva samples were obtained from a cohort of 279 (mean age: 56.5, SD: 13.0 years old, 51.5% male) COVID-19 survivors who needed hospitalization. Clinical data were collected from hospital medical records. Participants were asked to disclose pain symptoms developed during the first three months after hospital admission due to COVID-19 and persisting at the time of the interview. Methylations of five CpG dinucleotides in the *ACE2* promoter were quantified (as percentages). Participants were evaluated up to 17.8 (SD: 5.3) months after hospitalization. Thus, 39.1% of patients exhibited post-COVID-19 pain. Most patients (77.05%) in the cohort developed localized post-COVID-19 pain. Headache and pain in the lower extremity were experienced by 29.4% of the patients. Seven patients received a post-infection diagnosis of fibromyalgia based on the presence of widespread pain characteristics (11.6%) and other associated symptoms. No significant differences in methylation percentages at any CpG location of the *ACE2* promoter were identified when comparing individuals with and without post-COVID-19 pain. The current study did not observe differences in methylation levels of the *ACE2* promoter depending on the presence or absence of long-lasting post-COVID-19 pain symptoms in individuals who needed hospitalization due to COVID-19 during the first wave of the pandemic.

## 1. Introduction

The world has been immersed in the worst worldwide pandemic of the current century due to the rapid spreading of severe acute respiratory syndrome coronavirus 2 (SARS-CoV-2), the agent responsible for causing coronavirus disease 2019 (COVID-19). In addition to millions of deaths and billions of people infected with COVID-19 in the last four years, an important healthcare problem derived from SARS-CoV-2 infection has arisen in the potential development of long-lasting (or persisting) symptoms after an acute SARS-CoV-2 infection. The presence of symptoms once the acute COVID-19 phase has passed has received different names, such as long COVID, post-COVID-19, post-acute COVID-19 syndrome, and chronic post-COVID-19 [1]. A consensus paper proposed that the “post-COVID-19 condition occurs in people with a history of probable or confirmed SARS-CoV-2 infection, usually three months from the onset of infection, with symptoms that last for at least two months and cannot be explained by an alternative medical diagnosis. Common symptoms include, but are not limited to, fatigue, shortness of breath, and cognitive dysfunction, and generally have an impact on everyday functioning” [2].

Different meta-analyses have found that post-COVID-19 symptomatology can be present in up to 25–30% of subjects after recovery from an acute SARS-CoV-2 infection at one [3,4] and even two [5,6] years afterward. Additionally, the presence of post-COVID-19 symptomatology seems to be similar in comparisons between hospitalized and non-hospitalized COVID-19 survivors [3,4,5,6]. Although fatigue, dyspnea, or cognitive problems are usually reported as the most prevalent post-COVID-19 symptoms [3,4,5,6], pain is also a bothersome post-COVID-19 symptom, one that is experienced by 15–20% of post-COVID-19 survivors in the first six months after the acute infection [7]. A recent meta-analysis found that the prevalence of post-COVID-19 pain ranges between 8% to 17% during the first twelve months after COVID-19, although this prevalence rate depends on the study design, the definition of post-COVID-19 pain, and the outcomes used for collecting data [8]. Of particular relevance is that most published studies included in the two meta-analyses were not specifically focused on post-COVID-19 pain, and the reported prevalence rates are based on an examination of overall post-COVID-19 symptomatology [7,8]. In fact, the prevalence of post-COVID-19 pain has been found to be much higher, reaching up to 60%, when this symptom is specifically investigated [9,10,11,12].

Epigenetics has been proposed as one of the potential underlying mechanisms explaining post-COVID-19 pain [13]. Epigenetics include molecular processes that regulate gene expression without inducing changes in the DNA sequence [14]. Several epigenetic processes are described in the literature, methylation being one of the most investigated in COVID-19 research [15]. The potential effect of SARS-CoV-2 on epigenetics has been of interest from the beginning of the COVID-19 pandemic [16]. In fact, studies investigating epigenetics changes induced by SARS-CoV-2 infection are still being conducted [17]. Some studies have previously identified a heterogeneous response in methylation levels in COVID-19 patients at the acute phase of the infection; for instance, some genes such as interferon-related genes exhibited a hypermethylation (higher percentages) pattern, whereas other genes, such as those associated with the inflammatory response, exhibited a hypomethylation (lower percentages) pattern [18,19]. Thus, epigenetic changes in inflammatory-associated genes could explain the development of post-COVID-19 pain symptomatology. Balnis et al. [20] observed that those changes in methylation levels identified at the acute COVID-19 phase persisted at least one year after the infection in a small sample of 15 COVID-19 survivors. These results would suggest the possibility that epigenetics can potentially play a role in the development of post-COVID-19 symptomatology, particularly as to chronic pain. In fact, research work has focused on variations in the dynamics of DNA methylation in chronic pain conditions [21], but no study has specifically investigated DNA methylation changes and the presence of long-lasting post-COVID-19 pain symptomatology.

We have recently investigated the role of the DNA methylation levels of the angiotensin-converting enzyme 2 (*ACE2*) in the development of post-COVID-19 symptoms in individuals who needed hospitalization due to COVID-19 during the first wave of the outbreak [22]. The current paper presents a study, using the same cohort of patients [22], investigating whether DNA methylation levels of the *ACE2* promoter are associated with the development of long-lasting post-COVID-19 pain in individuals who had been hospitalized due to SARS-CoV-2 infection.

## 2. Methods of the Investigation

### 2.1. Participants

As described in the earlier paper [22], this study recruited subjects who were previously hospitalized at four urban hospitals in Madrid (Spain) due to COVID-19 during the first wave of the outbreak (March to May 2020). All included participants presented a confirmed positive diagnosis of SARS-CoV-2 infection as well as clinical/radiological findings at hospital admission. The study was approved by the Institutional Ethics Committees of all institutions (URJC0907202015920) and hospitals (HUFA 20/126; HUIL/092-20; HSO25112020; and HCSC20/495E) involved. Participants were informed of the study procedure, read the written informed consent, and signed it if they decided to participate in the study.

### 2.2. Genome DNA Collection

Evidence shows that using saliva to assess DNA methylation is becoming more common in the literature [23]. In fact, Khare et al. found that salivary DNA is equivalent in quantity and purity to blood DNA [24]. Accordingly, we used a saliva sample rather than a blood sample, because the former is a viable, non-invasive, and stress-free assessment method used to evaluate DNA methylation. In the experiment’s scenario, unstimulated whole saliva samples were collected during the morning hours from each patient, using collection tubes and following standardized procedures. Consistent with the manufacturer’s instructions, we asked participants to avoid eating, drinking or chewing gum for at least 1 h before saliva sample collection. After collection, samples were centrifuged at 3000 rpm for 15 min to obtain the cell sediment and stored at −20 °C until the DNA methylation analysis.

A MagMAX™ DNA Multi-Sample Ultra 2.0 Kit (Thermo Fisher Scientific Inc., Hemel Hempstead, UK) and King Fisher Flex purification robot (Thermo Fisher) were used for genomic DNA extraction. Purity and concentration of the resulting DNA were assessed using Quant-iT™ PicoGreen™ dsDNA reagent” (Thermo Fisher).

### 2.3. Differential Methylation Profiling

**Figure 1:**
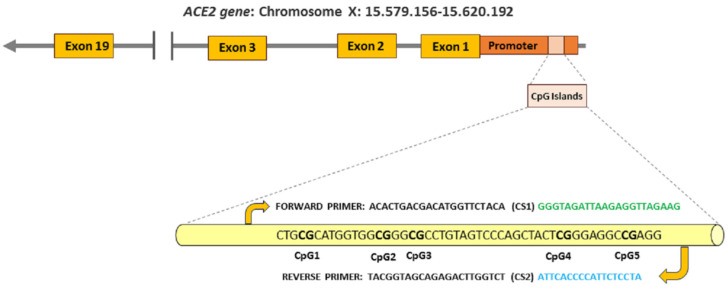
The figure graphs the forward (in green) and reverse (in blue) primers used for DNA methylation sequencing. In addition, each of the five CpG sites analyzed (CG in bold within the sequence) can be visualized in the overall island sequence associated with the *ACE2* promoter region.

We used the same procedures employed in our previous study [22]. Briefly, methylation percentages were calculated in five non-cytosine-phosphate-guanine (CpG) sites of interest within the *ACE2* promoter (CpG1, CpG2, CpG3, CpG4, and CpG5) as previously described [25,26]. The five CpG sites within the *ACE2* promoter were identified with a specific web-based program (http://www.urogene.org/methprimer, last accessed on 10 April 2024). Figure 1 graphs the CpG islands sequence in the promoter region of the *ACE2* receptor.

All methylation analyses procedures were carried out at Fundación Parque Científico de Madrid (FPCM), c/Faraday 7, Madrid, Spain, and have previously been extensively described [22]. For the main analyses, methylation percentage (%) at each position of the *ACE2* promoter (CpG1, CpG2, CpG3, CpG4, and CpG5) was used separately.

### 2.4. Data Collection

Age, gender, height, weight, pre-existing medical comorbidities, previous chronic pain conditions, days in hospital, COVID-19 onset-associated symptomatology, and need of intensive care unit (ICU) admission were collected from hospital medical records.

Included patients were scheduled for a face-to-face interview conducted by trained healthcare researchers with 15 years of experience in pain management. Thus, participants were asked about the presence of pain symptoms that appeared after their hospital stay due to SARS-CoV-2 infection, over at least the subsequent three months, in absence of any event explaining the developed of pain (e.g., trauma or surgery), and whether the pain persisted at each time of the study (consistent with the definition of a post-COVID-19 condition [2]). They were also asked to describe the location of their pain symptoms (e.g., head, cervical spine, shoulder, elbow–wrist, hip, knee, thorax, lower or upper extremity, or generalized pain). We used the definition of primary chronic musculoskeletal pain proposed by the International Association for the Study of Pain [27].

### 2.5. Statistical Analysis

The STATA software, version 16.1, was used for data collection, whereas the Python library pandas 0.25.3 was used for data processing. Quantitative data were expressed as means (standard deviations, SD), whereas the categorical data were expressed as numbers of cases (percentages). One-way ANOVA tests were used to determine differences in the methylation percentages (%) between patients with and without post-COVID-19 pain symptoms. The assumption of normality of the data was assessed with the Shapiro–Wilk test. A priori *p*-values lower than 0.05 were considered statistically significant; the Holm–Bonferroni correction for multiple comparisons was applied.

## 3. Results

As previously reported [22], a total of 330 individuals who needed hospitalization due to acute SARS-CoV-2 infection during the first wave of the COVID-19 pandemic were invited to participate. Fifty-one (15%) were excluded due to the following reasons: refusal to attend the appointment (n = 15), comorbid diagnosis of fibromyalgia (n = 15), DNA methylation analyses not possible due to contamination of the sample (n = 14), or pregnancy (n = 7). Ultimately, a total of 279 patients (51.3% male, mean age: 56.4 ± 12.8 years old) fulfilled all inclusion criteria.

**Figure 2:**
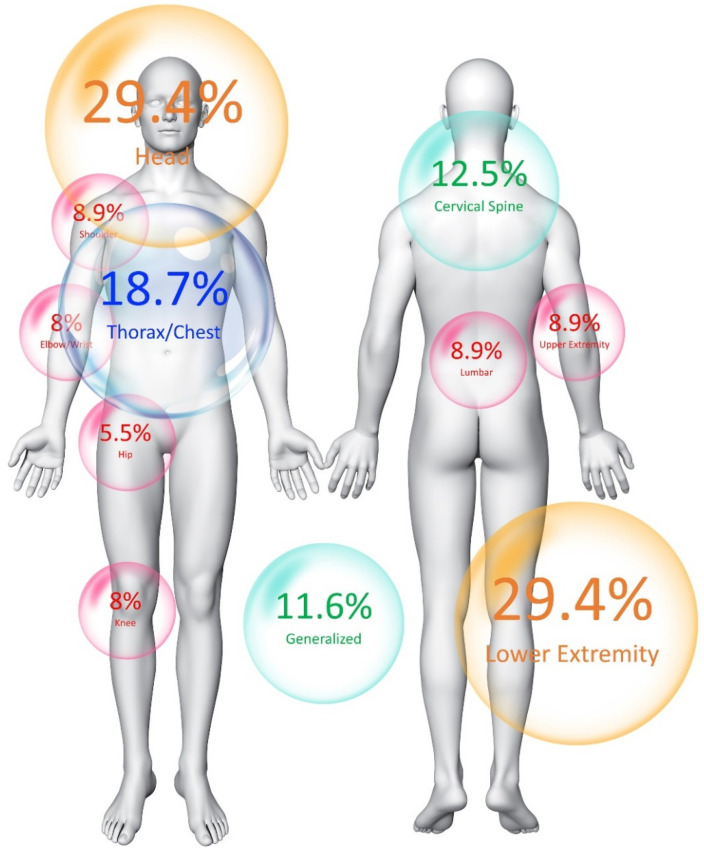
Location of post-COVID-19 pain symptomatology in the cohort analyzed (n = 109).

At the time of the study (mean: 17.8, SD: 5.2 months after hospital discharge), the prevalence of long-lasting post-COVID-19 pain symptomatology was 39.1% (n = 109). Most patients (77.1%) developed localized post-COVID-19 pain symptomatology. Thus, the location of post-COVID-19 pain symptoms is presented in Figure 2. Pain in the head and pain in the lower extremity were the most prevalent locations (29.4%). 

No significant differences in the presence of previous medical comorbidities were identified between patients who developed post-COVID-19 pain symptomatology and those who did not (Table 1). A significantly higher proportion of females reported post-COVID-19 pain (*p* = 0.005), when compared with males. Further, individuals who developed post-COVID-19 pain exhibited a higher number of COVID-19 onset-associated symptoms at hospitalization (*p* = 0.01), particularly COVID-19 onset-associated headache (*p* = 0.008, Table 1). No significant association was identified between the number of COVID-19 onset-associated symptoms at hospitalization and methylation levels at CpG1 (r = 0.021, *p* = 0.829), CpG2 (r = 0.026, *p* = 0.787), CpG3 (r = 0.061 *p* = 0.524), CpG4 (r = 0.017, *p* = 0.861), or CpG5 (r = 0.091, *p* = 0.340).

Overall, no significant differences in methylation percentages in any of the CpG locations of the *ACE2* promoter were identified when comparing COVID-19 survivors who developed post-COVID-19 pain symptoms and those who did not (Table 1). The mean intensity of post-COVID-19 pain was 5.6/10 (SD: 1.7) points. No significant association existed between the intensity of post-COVID-19 pain and methylation levels at CpG1 (r = 0.06, *p* = 0.959), CpG2 (r = 0.187, *p* = 0.101), CpG3 (r = 0.078 *p* = 0.496), CpG4 (r = 0.111, *p* = 0.325), or CpG5 (r = 0.175, *p* = 0.184). Similarly, no significant association was observed between the length of pain symptoms and methylation levels at CpG1 (r = 0.11, *p* = 0.912), CpG2 (r = 0.083, *p* = 0.381), CpG3 (r = 0.124 *p* = 0.193), CpG4 (r = 0.011, *p* = 0.905), or CpG5 (r = 0.115, *p* = 0.228).

No differences as to the presence of previous chronic pain conditions were identified when comparing the presence or absence of post-COVID-19 pain (Table 2). Seven (6.4%) patients received a diagnosis of fibromyalgia syndrome based on the presence of widespread pain (11.6%) and other associated symptoms. Finally, five (4.6%) and twenty-seven (24.8%) patients received diagnoses of migraine and tension-type headache, respectively (Table 2).

## 4. Discussion

The present study investigated the potential correlation between methylation levels in the promoter of the *ACE2* gene and the development of long-lasting post-COVID-19 pain symptoms over one-and-a-half years in patients who need hospitalization due to COVID-19 during the first wave of the pandemic. Several studies have highlighted the roles of the surface receptor for S1 of the *ACE2* and the transmembrane protease serine-2 (TMPRSS2) receptor in subjects during the acute COVID-19 phase [28]. It is known that SARS-CoV-2 enters the host cells through the membrane-bound *ACE2* exopeptidase, and hypomethylation of *ACE2* may potentially increase its expression, thereby elevating the risk of infection [29] and accordingly elevating the risk of post-COVID-19 condition. The results obtained in this cohort of COVID-19 survivors did not show a significant correlation between this specific gene investigated and the development of post-COVID-19 pain symptomatology.

### 4.1. Post-COVID-19 Pain and DNA Methylation Changes

The prevalence of pain symptoms in our cohort of previously hospitalized COVID-19 survivors at a follow-up of 18 months after the infection was 40%. This prevalence rate is higher than those found in published meta-analyses, including studies investigating overall post-COVID-19 symptomatology (including pain) and reporting that 8% to 20% of COVID-19 survivors exhibit post-COVID-19 pain the first year after the infection [7,8], but it is lower in comparison with studies specifically investigating the prevalence of post-COVID-19 pain, where prevalence rates reach to up to 60% of the patients [9,10,11,12]. Thus, it is remarkable that most published studies included follow-up periods shorter than one year [7,8,9,10,11,12]. Since the prevalence of post-COVID-19 pain symptomatology (and also the overall post-COVID-19 condition) tends to decrease with time [30], prevalence data from our sample can be considered representative of this population.

No previous study has investigated DNA methylation changes in individuals with post-COVID-19 pain. It seems that post-COVID-19 pain is associated with the inflammatory response related to COVID-19 [31]. Thus, the fact that individuals who report myalgia as an associated symptom at the acute COVID-19 phase are at a higher risk of developing post-COVID-19 pain [32] supports the finding that muscle pain is specifically sensitive to the cytokine SARS-CoV-2-associated burst. Nevertheless, it has also been reported that long-term post-COVID-19 myalgia is associated with lower levels of inflammatory biomarkers (e.g., interleukins-6) at the acute COVID-19 phase [33].

Interestingly, DNA methylation changes at CpG sites of specific pain genes such as *OPRM1* (opioid receptor Mu 1) and *TRPA1* (Transient Receptor Potential Cation Channel Subfamily A Member 1) have been associated with sensitivity to pain [34]. Therefore, this association could be post-COVID-19-symptom-specific. For instance, Takenaka et al. [35] have observed an association between methylation levels of the *TRPA1* gene promoter region and the presence of neuropathic-like symptoms [35]. Hence, it is possible that the presence of post-COVID-19 pain symptomatology can be associated with DNA methylation changes in genes associated with inflammation, (e.g., *OPRM1* or *TRPA1*) rather than in those genes associated with COVID-19 susceptibility (e.g., *ACE2* promoter) such as those described in our study.

Finally, it is also important to understand that no timeframe can be determined for DNA methylation change identification. In fact, no longitudinal study has investigated those variations of DNA methylation at different timeframes. Thus, it can be hypothesized that COVID-19 could induce different DNA methylation changes at the acute phase of the infection, while these changes reverse afterward. Future studies investigating the longitudinal evolution of DNA methylation changes from the acute COVI-19 phase to the development of post-COVID-19 pain in the context of long-term follow-ups are needed.

### 4.2. DNA Methylation and Widespread Pain

Among those patients developing post-COVID-19 pain, of particular interest are those developing widespread pain symptomatology, like fibromyalgia syndrome [36,37]. Individuals with widespread pain exhibit nociplastic pain features, which means that these patients need particular medical attention due to the complexity of their clinical presentation [38]. Previous studies have explored DNA methylation changes in patients with chronic widespread pain [39] or fibromyalgia syndrome [40], providing evidence that DNA methylation alterations can be relevant in widespread pain conditions. In the current study, thirteen (11.6%) individuals reported widespread pain symptoms. Among these patients, seven (6.4%) had received a diagnosis of fibromyalgia syndrome one year after the infection. In fact, it has been suggested that SARS-CoV-2 could act as a trigger factor of fibromyalgia syndrome, or as an exacerbator factor, since both conditions share similar mechanisms [41]. Thus, we conducted a secondary analysis looking to see whether COVID-19 survivors with widespread post-COVID-19 pain symptomatology (n = 13) exhibited different DNA methylation percentages than those reporting localized post-COVID-19 pain (n = 96). No significant differences in methylation percentages in any of the CpG sites were seen (Table 3).

It is possible that the small sample size of the subgroup of patients with widespread pain symptoms (n = 13) did not permit the detection of significant differences, although this is unlikely. Additionally, it is also possible that DNA methylation changes are gene-specific, since patients with chronic fatigue syndrome and fibromyalgia syndrome are mainly characterized by altered DNA methylation in those genes regulating cellular signaling and immune functioning [42]. Nevertheless, we should recognize that we did not phenotype the type of pain symptomatology and were not able to determine if the symptoms had a nociceptive, neuropathic, or nociplastic pain phenotype.

### 4.3. Previous Pain Conditions

It has been previously seen that a suffering from musculoskeletal pain before an acute SARS-CoV-2 infection increases the risk (OR1.55, 95%CI 1.27 to 1.89) of post-COVID-19 pain [43]. This finding was confirmed in a large retrospective study determining that the presence of chronic pain conditions before SARS-CoV-2 infection increases the risk of post-COVID-19 pain symptomatology [44]. Although the prevalence of previous chronic pain conditions was higher in COVID-19 survivors who developed post-COVID-19 pain than among those who did not develop pain, the differences were not statistically significant in our study.

### 4.4. Female Sex

Female sex has been found to be a risk factor associated with overall post-COVID-19 condition [45,46] and also specifically with reference to post-COVID-19 pain [43]. In our cohort, we also saw that the proportion of females reporting post-COVID-19 pain was significantly higher than that of the males. This result could be expected since musculoskeletal pain is more prevalent in females than in males [47,48]. Several biological and sociocultural factors, as well as gender-constructed behaviors, have been proposed as bases for explaining sex differences in COVID-19 and post-COVID-19 responses [49]. An important biological factor associated with the current study is that the expression of the *ACE2* receptor is more pronounced in males than in females, since estrogens can down-regulate its expression [50]. This factor could provide a plausible biological explanation for the reduced severity of COVID-19 in females, but it would not explain the higher prevalence of post-COVID pain in females.

### 4.5. Limitations

Although this is the first study investigating DNA methylation changes at the *ACE2* promoter and the development of long-lasting post-COVID pain symptomatology, some limitations must also be recognized. First, we included a cohort of patients who need hospitalization when they were infected with a historical SARS-CoV-2 strain; therefore, extrapolation of the current results to other populations should not be attempted. In addition, the sample size could be considered relatively small. Second, the cross-sectional design of our study does not permit the determination of the fluctuating nature of DNA methylation changes. Third, we only analyzed DNA methylation changes at the *ACE2* promoter; hence, we cannot exclude the presence of DNA methylation alterations in pain-associated genes. Finally, we did not collect pain features associated with our sample, so proper characterization of post-COVID pain was not conducted. Therefore, studies including large samples of individuals, hospitalized due to COVID-19 and non-hospitalized, and including whole DNA methylation analyses, might be able to identify epigenetic changes associated with the development of long-lasting post-COVID pain symptomatology.

## 5. Conclusions

The results from the current study did not find an association between the methylation levels at different CpG sites of *ACE2* promoter and the development of post-COVID pain symptomatology in the one-and-a-half years after suffering from COVID-19 in a cohort of individuals who needed hospitalization due to the infection. Future studies investigating multiple sites where, after infection by SARS-CoV-2, methylation of CpG might more specifically regulate the pain pathways are needed.

## Figures and Tables

**Table 1:** Demographic, clinical, and methylation percentage of individuals with and without post-COVID-19 pain.

	Post-COVID-19 Pain (n = 109)	No Post-COVID-19 Pain (n = 170)	*p* Value
Age, mean (SD), years	55.7 (12.2)	57.0 (13.2)	0.443
Sex, male/female (%) *	40 (46.7%)/69 (63.3%)	103 (61.6%)/67 (39.4%)	0.005 *
Weight, mean (SD), kg	80.1 (17.1)	81.6 (16.6)	0.447
Height, mean (SD), cm	167.5 (9.3)	169.0 (9.6)	0.159
Previous medical pathologies (n)	1.25 (1.0)	1.35 (1.0)	0.286
Previous medical pathologies			
Hypertension	36 (33.0%)	59 (34.7%)	0.815
Diabetes	14 (12.8%)	15 (8.8%)	0.309
Cardiovascular Diseases	6 (5.5%)	14 (8.25%)	0.406
Asthma	15 (13.7%)	16 (9.4%)	0.288
Obesity	38 (34.8%)	47 (27.7%)	0.287
Chronic Obstructive Pulmonary Disease	1 (0.9%)	4 (2.35%)	0.382
Number of symptoms associated with COVID-19 at hospital admission, mean (SD) *	3.4 (0.8)	3.1 (1.1)	0.01 *
COVID-19 symptoms at hospitalization			
Fever	36 (70.6%)	125 (73.5%)	0.782
Dyspnea	44 (41.3%)	57 (33.5%)	0.295
Myalgia	42 (38.5%)	74 (43.5%)	0.105
Cough	45 (42.2%)	54 (31.8%)	0.347
Headache *	46 (42.2%)	41 (24.1%)	0.008 *
Diarrhea	19 (17.4%)	35 (20.6%)	0.558
Anosmia	21 (19.3%)	42 (24.7%)	0.351
Ageusia	22 (20.2%)	43 (25.3%)	0.388
Throat Pain	12 (11.0%)	20 (11.7%)	0.855
Vomiting	10 (9.2%)	13 (7.6%)	0.664
Dizziness	6 (5.5%)	10 (5.9%)	0.897
Intensive Care Unit (ICU) admission			
Yes/No, n (%)	4 (3.7%)/105 (96.3%)	6 (3.5%)/164 (96.5%)	0.905
CpG1 methylation (%)	93.4 (4.3)	93.6 (3.4)	0.608
CpG2 methylation (%)	40.1 (7.2)	40.0 (7.5)	0.881
CpG3 methylation (%)	43.8 (8.5)	43.0 (8.8)	0.445
CpG4 methylation (%)	45.4 (8.0)	45.7 (7.9)	0.784
CpG5 methylation (%)	0.6 (0.3)	0.6 (0.7)	0.971

n: number; SD: standard deviation; * Statistically significant differences between groups (*p* < 0.05).

**Table 2:** Chronic pain condition diagnoses of individuals with and without post-COVID-19 pain.

	Post-COVID-19 Pain (n = 109)	No Post-COVID-19 Pain (n = 170)	*p* Value
Pre-COVID-19 Chronic Pain Conditions			
Chronic Pain Symptomatology	60 (55.0%)	70 (41.2%)	0.10
Migraine	10 (9.2%)	7 (4.1%)	0.09
Tension-Type Headache	14 (12.8%)	12 (7.1%)	0.122
Rheumatoid Arthritis	3 (2.75%)	6 (3.5%)	0.724
Osteoarthritis	17 (15.6%)	16 (9.4%)	0.143
New Post-COVID-19 Chronic Pain Conditions			
Localized Pain	84 (77.05%)	----	----
Migraine	5 (4.6%)	----	----
Tension-Type Headache	27 (24.8%)	----	----
Fibromyalgia Syndrome	7 (6.4%)	----	----
Osteoarthritis	4 (3.7%)	----	----

n: number.

**Table 3:** Methylation percentages in individuals with and without post-COVID-19 widespread pain.

	Widespread Pain (n = 13)	Localized Pain (n = 96)	*p* Value
CpG1 methylation (%)	93.8 (2.8)	93.4 (3.8)	0.703
CpG2 methylation (%)	39.7 (7.0)	40.0 (7.4)	0.860
CpG3 methylation (%)	42.8 (7.5)	43.3 (8.7)	0.841
CpG4 methylation (%)	44.2 (8.4)	45.6 (7.8)	0.527
CpG5 methylation (%)	0.65 (0.3)	0.6 (0.35)	0.739

## Data Availability

The original contributions presented in the study are included in the article, further inquiries can be directed to the corresponding author.
